# Atypical reward anticipation in impulsive individuals: evidence from EEG and experiential delay discounting

**DOI:** 10.3389/fpsyg.2025.1746734

**Published:** 2026-01-12

**Authors:** Felix Zakirov, Olga Sysoeva

**Affiliations:** 1 Institute of Higher Nervous Activity and Neurophysiology of the Russian Academy of Sciences, Moscow, Russia; 2 Center for Cognitive Sciences, Sirius University of Science and Technology, Sochi, Russia

**Keywords:** delay discounting, EEG, experiential delay, impulsivity, time-frequency analysis, reward anticipation

## Abstract

**Background:**

Impulsivity is characterized by impaired inhibition of thoughts and actions and a reduced ability to weigh the consequences of behavior. Importantly, impulsivity has been associated with altered reward processing and reward anticipation.

**Methods:**

We aimed to investigate neurophysiological indices of impulsive behavior using an experiential delay discounting task with 64-channel EEG in neurotypical adults (*n* = 54). Impulsive behavior was operationalized as the preference for smaller, immediate rewards over larger, delayed rewards with the delay varied from 1 to 30 s in different blocks.

**Results:**

We registered feedback-related fronto-central theta and centro-parietal delta power, although did not find any correlation between impulsive behavior and these neurophysiological measures of reward sensitivity. However, we found higher power spectral density in the alpha band over parieto-occipital region during reward anticipation in individuals exhibiting more impulsive behavior in our task.

**Conclusion:**

These results suggest that impulsive individuals have less efficient mechanisms of reward anticipation, potentially resulting in lower tolerance for waiting.

## 1 Introduction

Impulsive behavior is characterized by an impaired ability to inhibit thoughts or actions and to weigh the consequences of one’s behavior (Moeller et al., 2001). Importantly, impulsive behavior is a hallmark of multiple mental health disorders, such as substance use disorder (Kozak et al., 2019), gambling disorder (Ioannidis et al., 2019), and attention-deficit/hyperactivity disorder (ADHD) (Winstanley et al., 2006). Notably, impulsivity is a multifaceted construct that comprises different domains (Kozak et al., 2019) that do not always correlate with each other (Calluso et al., 2015; Friedman et al., 2020; Hedge et al., 2020). Thus, the mechanisms of impulsive behavior remain unclear, with studies suggesting they can be driven by impaired integration between regions involved in rewards evaluation and emotion regulation (Kerr et al., 2015), atypical processing of the waiting period (Scheres et al., 2007; Ströhle et al., 2008; Beck et al., 2009) or enhanced reward sensitivity (Herbort et al., 2016; Mechelmans et al., 2017).

With the use of methods that allow for non-invasive measurement of brain activity, such as electroencephalography (EEG), neural markers of reward processing and reward anticipation can be identified and compared across individuals. For example, a well-established electrophysiological measure of anticipatory activity is the decrease of power in the alpha (8–12 Hz) band over parieto-occipital regions, which has been linked to sustained attention (Bacigalupo and Luck, 2022) and inhibition of distractors (Jensen and Mazaheri, 2010; Doesburg et al., 2016; Woodman et al., 2022). Importantly, alpha power has been shown to decrease in response to cues preceding rewards (van den Berg et al., 2014) and is associated with a preference for larger delayed rewards (Pornpattananangkul and Nusslock, 2016). This suggests that a decrease in power in the alpha band may be an important index of the efficiency of reward anticipation. Additionally, there are consistently reported electrophysiological markers of reward processing, such as feedback-related increases in power in the delta (1–3 Hz) and theta (4–7 Hz) bands. For example, an increase in delta band power has been consistently reported in response to gains compared to losses (Nelson et al., 2017; Mei et al., 2018; Ethridge et al., 2020) and is sensitive to the magnitude of rewards (Watts and Bernat, 2018). Finally, feedback-related theta is another important oscillatory marker of reward processing that has also been shown to be sensitive to the magnitude of rewards (Paul et al., 2020) and to track cognitive control demands required for successful task performance (Cavanagh and Frank, 2014) and receiving rewards (Mei et al., 2018).

Delay Discounting (DD), also known as temporal discounting, is a widely described paradigm to study impulsivity, which manipulates the delay duration required to receive a larger rewards (Green et al., 1996; Story et al., 2014). In this regard, impulsive behavior manifests as a preference for a smaller but sooner reinforcement over a larger but delayed one (da Matta et al., 2012). Crucially, DD enables the study of not only the reward processing stage of decision-making but also reward anticipation. Existing studies utilizing DD with different designs have provided insights into impulsive decision-making in various contexts, such as food behavior (Skrynka and Vincent, 2019; Epstein et al., 2023), substance abuse (Yi et al., 2010; Robles et al., 2011), and normative behavior (Schwenke et al., 2022), suggesting that DD is a reliable paradigm for assessing the temporal discounting domain of impulsivity. To date, existing studies investigating DD using EEG have used designs with monetary incentives and very large delays ranging from weeks to months (Pornpattananangkul and Nusslock, 2016; Patalano et al., 2020; Wang et al., 2020) that cannot be directly experienced by participants in the form of reward anticipation. Moreover, monetary rewards, although studied more often, are not necessary to induce reward-related brain activity (Distefano et al., 2018; Tunison et al., 2019). Thus, there is a need for electrophysiological studies on DD with experiential, rather than hypothetical, delay and non-monetary incentives. Furthermore, such experiential design bridges the gap between human and animal studies, as only experiential delays can be used for the latter (Lukinova et al., 2019).

In this study, we used an experiential DD task with non-monetary rewards and EEG to investigate the electrophysiological correlates of impulsive decision-making. We hypothesized that individuals who make impulsive choices in the delay-discounting task would have increased sensitivity to rewards, as indexed by increased feedback-related power in the delta and theta bands. Additionally, we hypothesized that the preference for smaller, immediate rewards in impulsive decision-makers would be associated with a deficiency in reward anticipation, as indexed by relatively higher alpha band power in the parieto-occipital regions during the delay.

## 2 Materials and methods

### 2.1 Participants

A total of 54 healthy adults (mean age = 25.50, SD = 6.14, 11 males, 43 females) participated in the study. Participants had no history of neuropsychiatric disorders and were not taking any psychopharmacological medications. The sample size for our study was determined using G*Power 3.1 (Faul et al., 2009) to be sufficient to detect the hypothesized effects of medium to large size (Cohen’s f2 = 0.2), as demonstrated in previous similar studies (Kamarajan et al., 2008; Leicht et al., 2013; Pornpattananangkul and Nusslock, 2016), with alpha error probability of 0.05 and 80% power.

### 2.2 Recording and procedure

During the experimental session, participants sat in a comfortable chair in a quiet room with dim lighting. The task was presented on a 24” LCD screen with a 120 Hz refresh rate. EEG data were obtained using a 64-channel Brain Products setup (actiCHamp Plus, Brain Products GmbH, Gilching, Germany). A standard 10-10 montage with an online reference at FCz and a ground electrode placed at FPz was used. The data were sampled at a rate of 500 Hz.

### 2.3 Task

**FIGURE 1:**
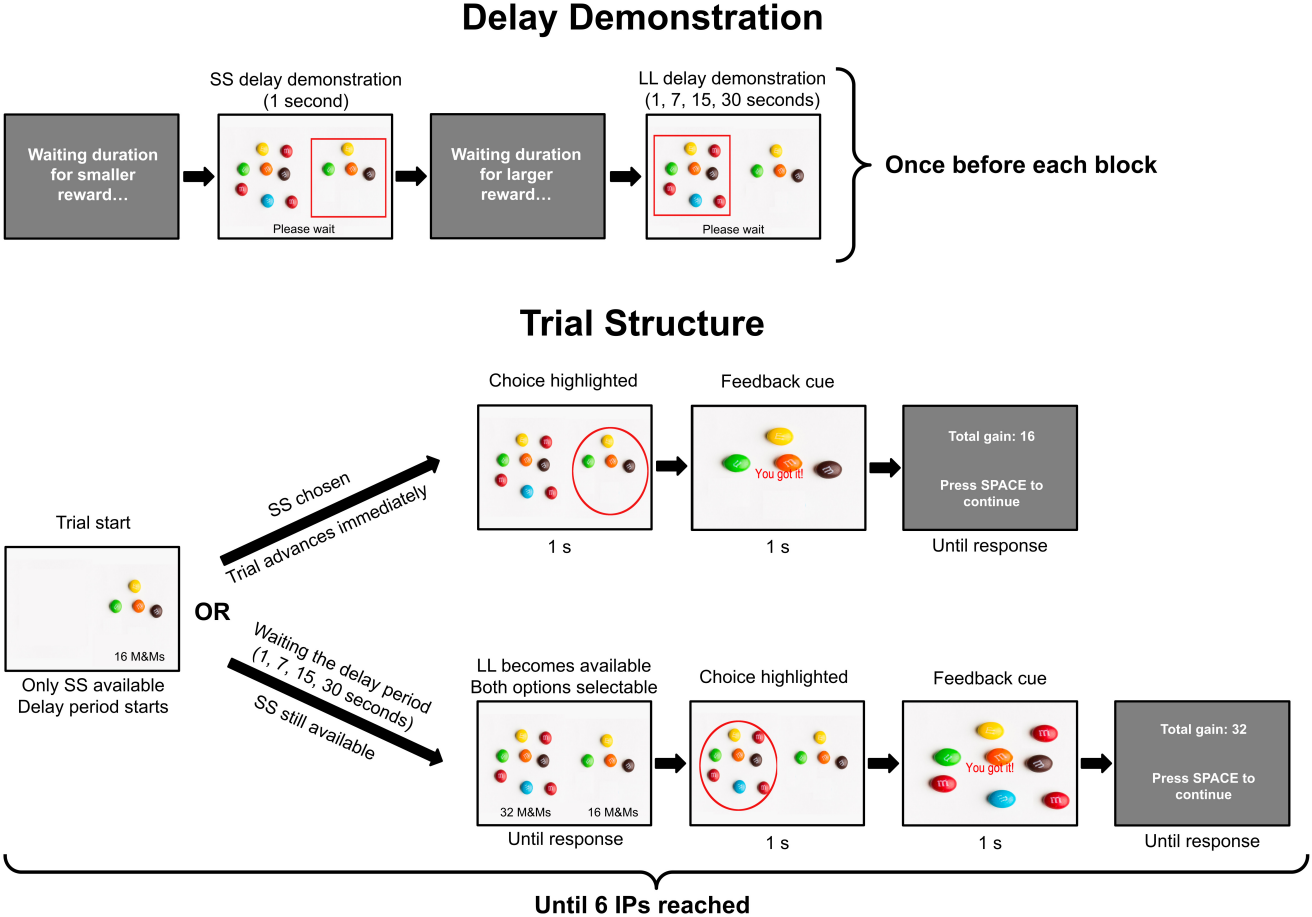
Experimental paradigm depiction. The first line shows a presentation of the delay period that was demonstrated once before each block. Each trial of the main blocks started with the presentation of SS, which became available for selection immediately after the onset of the trial. Its presentation also marked the beginning of the delay period. LL was not displayed and was unavailable for choosing until its associated delay period elapsed. Importantly, if a participant selected the SS option at any point during the delay period, the delay was immediately aborted, and there was no post-choice enforced waiting period. If the participant refrained from choosing SS and waited for the full duration of the delay, LL appeared alongside the SS option. After that moment, participants could choose this option by pressing the associated button. In both scenarios, the selected option (SS or LL) was highlighted with a red circle for 1 s, and the trial moved to the 1-s feedback cue, after which the cumulative score was presented before initiating the next trial. Note that the image displayed in the feedback cue depended only on the selected option (SS or LL) and not on the duration of the delay period in a given task block. IP, indifference point (see the main text).

An experiential DD task [adapted from Steele et al. (2019)] (Figure 1) was used in this study and developed using PsychoPy software (Peirce et al., 2019). Participants were instructed to play a game with the objective of maximizing their cumulative points represented as M&M’s candies, which were operationalized as non-consumable visual cues The use of candy imagery served to “gamify” the point accumulation, distinguishing it from standard monetary cues, but they remained hypothetical in nature. Furthermore, M&Ms also represent a palatable food stimulus, which is typically difficult to resist when readily available (French et al., 2001). The participants were informed that both the number of trials and game duration were fixed and unaffected by their choices, although it was not fully correct, as the duration of the experiment depended on the complex algorithm of the stair case procedure, which will be described below. This instruction served as a necessary methodological control designed to prevent participants from prematurely terminating the experiment. Given that we did not offer monetary compensation commensurate with the points collected, this instruction was intended to limit the number of participants who might speed through the experiment (by not choosing the larger delayed rewards, see below) and fail to pursue the stated goal of maximizing points.

On each trial, participants selected between two options: an immediate but smaller rewards (16 candies) or a larger but delayed rewards (32 candies) by pressing “1” or “2” on a standard QWERTY keyboard. The rewards were presented as a picture of M&Ms, with reward magnitudes additionally displayed beneath each option. For convenience, we will refer to these reward options as SS (small/sooner) and LL (large/later) further throughout the text. Before the four blocks of the main task, participants completed a 4-trial practice block to familiarize themselves with the key mapping and the two reward types (SS and LL). Trials in the practice block were identical to those in the main task, with the exception that the LL delay was 3 s. Each trial started with the presentation of SS, which became available for selection 1 s after the onset of the trial. The option for the LL reward was not displayed and was unavailable until its associated delay period elapsed. Importantly, if a participant selected the SS option at any point during the delay period, the delay was immediately aborted, and there was no post-choice enforced waiting period. If the participant refrained from choosing SS and waited for the full duration of the delay, LL appeared alongside the SS option and became available for selection. In both scenarios, the selected option (SS or LL) was highlighted with a red circle for 1 s and the trial moved to the 1-s feedback cue, after which the cumulative score was presented before initiating the next trial. Thus, the experimental design required participants to actively inhibit the immediately available SS option to secure the opportunity to choose the LL reward. We hypothesized that this requirement would make the waiting period difficult to tolerate for impulsive individuals, despite the virtual, non-monetary nature of the incentives, consistent with prior evidence showing that time preferences remain reliable across widely differing paradigms, including those utilizing immediate, symbolic reinforcers rather than real consumable rewards (Lukinova et al., 2019).

The entire game comprised four consecutive blocks, each with a different delay for the LL, presented in the following order: 1, 15, 30, and 7 s. At the beginning of each block, participants experienced the full delay duration as a demonstration of the delay period, separately for both SS and LL (Figure 1). Option positions (left/right) were counterbalanced across trials.

In our task, a staircase procedure was applied to adjust the amount of the SS. The SS amount gradually decreased (from initial 16 to a minimum of 1 candy) in subsequent trials if chosen, and gradually increased (to a maximum of 30 candies) if the LL was chosen. The amount of the LL remained constant throughout the task. Block termination occurred when either six indifference points (IPs) were reached within the block or the participant reached the minimum (1 candy) or maximum (30 candies) for the SS and maintained this preference for six consecutive choices, indicating a stable preference for a given reward option. IPs were calculated as the mean SS value across three consecutive trials featuring alternating choices (e.g., SS-LL-SS or LL-SS-LL), representing subjective equivalence between options.

The mean of the six IPs for each of four blocks, normalized by the maximum value reached for the SS within these blocks, was used to compute the discounting factor (DF). The DF served as an indicator of the strategy individuals adopted in the task (impulsive or self-controlled). The DF was calculated as the area under the curve (AUC) connecting the mean normalized IPs for each delay (Odum, 2011; Yeh et al., 2021). Accordingly, if a participant consistently chose the LL reward, the SS amount gradually increased, resulting in an increase in the DF (with a maximum DF of 1). Conversely, if the participant chose the SS, its amount gradually decreased, resulting in a decrease in the DF (with a minimum DF of 0).

Symbolic performance-contingent bonuses (a cup of coffee, a marshmallow, or a piece of cake) were awarded for reaching cumulative point thresholds (200, 400, or 600 points, respectively), with these thresholds visibly displayed during the task. Upon completion of all four blocks, participants viewed their total accumulated points, marking the end of the game.

### 2.4 Data processing

Electroencephalography preprocessing was performed using MNE Python (Gramfort et al., 2013; Larson et al., 2023). A 0.1–40 Hz band-pass filter was applied, and the data were re-referenced to the average activity across all electrodes. The data underwent visual inspection for bad channels and muscle artifacts. Independent component analysis (ICA) was performed, and artifacts were manually removed with reference to the ALICE algorithm (Soghoyan et al., 2021). All epochs exceeding a ±150 μV amplitude threshold were rejected (Mognon et al., 2011). For time-frequency analysis, the data were resampled to 250 Hz. Feedback-locked epochs were extracted in the [−1, 2 s] time window, with zero corresponding to the onset of the feedback cue (Figure 1). For reward anticipation epochs, the delay period (7, 15, or 30 s), beginning with the delay period (Figure 1), was segmented into 1-s epochs with 50% overlap. Time-frequency decomposition was performed using Morlet wavelets for frequencies between 1 and 35 Hz. Prior to averaging, time-frequency epochs were baseline-corrected using a window from −300 to −100 ms to prevent leakage of post-stimulus activity into the baseline period, which may occur at lower frequencies (Cohen, 2014).

Feedback-locked theta power (4–7 Hz) was computed at the fronto-central cluster (Fz, FC1, FC2) in a 200–400 ms window. Feedback-locked delta power (1–3 Hz) was computed at the centro-parietal cluster (Cz, CPz) for a 100–600 ms window. The time windows for feedback-related time-frequency measures were selected based on inspection of grand-average waveforms and previous studies (Pornpattananangkul and Nusslock, 2016; Paul and Pourtois, 2017; Ethridge et al., 2020). Due to the lower trial count for SS choices, only epochs for LL choices were included in the analysis. This ensured a sufficient number of epochs per participant after exclusion of two participants with an epoch number lower than 12 (*n* = 52, *M* = 27.52, SD = 8.09, range = 12–39).

In the main analysis of anticipatory alpha-band activity, only participants with at least one artifact-free trial in the 7-, 15-, or 30-s block were included (*n* = 52). Anticipatory alpha band (8–12 Hz) power spectral density (PSD) was computed at the parieto-occipital electrode site (POz) (Bacigalupo and Luck, 2022) as the average of corresponding 1-s epochs (number of epochs per participant per condition: *M* = 302.40, SD = 114.33, range = 29–539). Such an approach allowed us to normalize for different durations of the delay as well as to mitigate a low trial count. Thus, the minimum number of epochs contributed by any participant was 29, which exceeds the threshold previously shown to yield reliable EEG estimates (Pontifex et al., 2010).

### 2.5 Statistical analysis

The statistical analysis was performed in R (R Core Team, 2021) and included two generalized linear models (GLM) (relationship between DF and feedback-locked delta, and theta power) and a linear mixed model (LMM) (between DF and alpha PSD), with a random intercept fit for each participant. The use of LMM for the alpha PSD data, which contained multiple levels of delay per participant, ensured the robustness of our analysis to potential missing and unbalanced data and to asymmetrical distributions (Schielzeth et al., 2020). Prior to statistical modeling, outliers exceeding ±3 SD from the mean were removed.

The GLMs s included one continuous between-subject predictor, DF. The LMM for the anticipatory alpha band PSD included one categorical within-subject predictor, the duration of the delay (7/15/30 s), and one continuous between-subject predictor (DF).

If a significant main effect between groups was found in the full models, *post-hoc* tests adjusted for false discovery rate (FDR) (Benjamini and Hochberg, 1995) were conducted. If a significant continuous-categorical interaction was found in the full models, simple slopes were computed.

Additionally, we conducted a complementary analysis to examine the relationships between DF and task duration using Spearman’s correlation coefficient.

## 3 Results

### 3.1 Behavioral results

To characterize the overall trend for the strategy participants followed in the DD task, we first present the descriptive statistics of DF. On average, participants tended to choose larger delayed (LL) rewards over smaller, immediate (SS) rewards, with a mean DF of 0.825 (SD = 0.226). Accordingly, the DF distribution in our sample was negatively skewed. The negative skewness of DF was likely caused by the instructions, which encouraged the majority of participants to consistently choose the LL option (as maximizing points was the explicit goal and task duration was perceived as fixed), thereby contributing to the observed ceiling effect. The mean duration of the task was 15.475 min (SD = 3.6). Our complementary analysis showed a significant positive correlation between DF and task duration (*r* = 0.586, *p* < 0.001). A visual depiction of the averaged DD curve DF distribution, as well as the relationships between the task duration and DF, is provided in the Supplementary Figures 1, 2.

### 3.2 Feedback-related theta and delta power

**FIGURE 2:**
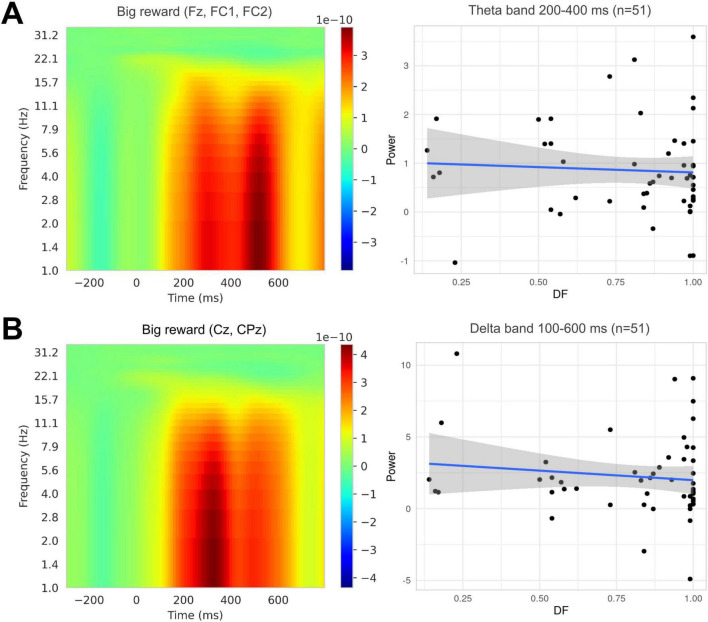
**(A)** Feedback-related theta at the frontal-central cluster (Fz, FC1, FC2), *n* = 51. **(B)** Feedback-related delta at the centroparietal cluster (CPz, Pz), *n* = 52. None of the measures showed a significant relationship with DF (*p* = 0.679 and *p* = 0.391, respectively). The regression line depicts the linear model fit, and the shaded region indicates the 95% confidence interval.

As we hypothesized that impulsive behavior would be associated with increased sensitivity to rewards, we examined the time-frequency measures of reward processing. We observed feedback-related increases in theta and delta band power in our task, as can be seen in Figure 2. However, neither band’s activity within the predefined time windows was related to individual strategy in the DD task. In the model with feedback-related delta power as the dependent variable, we did not find a significant effect of DF [F(1, 49) = 0.748, *p* = 0.391]. Similarly, we did not find a significant effect of DF on feedback-related theta power [F(1, 49) = 0.173, *p* = 0.679]. Therefore, in contrast to our hypotheses, none of the feedback-related measures showed significant associations with impulsive behavior in the DD task.

### 3.3 Anticipatory alpha PSD

**FIGURE 3:**
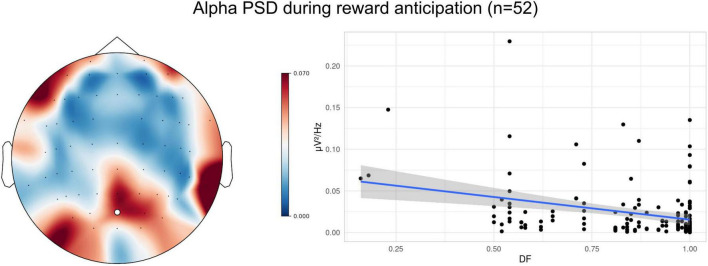
Averaged parieto-occipital alpha band PSD (*n* = 52) during the LL anticipation across three delay levels (7, 15, 30 s). Individuals with higher DF (more self-controlled) exhibited reduced alpha PSD during reward anticipation. The regression line depicts the linear model fit, and the shaded region indicates the 95% confidence interval.

In line with the original hypothesis, we tested the association between DF and reward anticipation indexed by the parieto-occipital alpha band. The full model with parieto-occipital alpha band PSD indicated a significant main effect of DF [F(1, 66) = 11.934, *p* = 0.001). In particular, individuals with higher DF (more self-controlled) exhibited reduced alpha PSD during reward anticipation (Figure 3). No main effect of delay [F(2, 110) = 2.014, *p* = 0.138] or delay*DF interaction [F(2, 109) = 0.601, *p* = 0.550] was found. For completeness, we also provide a figure depicting alpha PSD-DF relationships for each level of the delay, although they were qualitatively similar across all delay levels (Supplementary Figure 3).

## 4 Discussion

In this study, we investigated two processes underlying impulsive decision-making, as indexed by the delay discounting (DD) task: reward-related feedback processing and reward anticipation in the context of an experiential DD task with non-monetary rewards. Our main findings showed that individuals exhibiting greater self-control demonstrated reduced alpha band power during reward anticipation. Additionally, we showed that reward processing, as measured by feedback-related theta and delta power, did not correlate with impulsivity as measured by the experiential DD task.

One of the key characteristics of impulsivity, as reported at the trait (Torrubia et al., 2001), behavioral (Robles et al., 2011; Jones et al., 2015), and neural (Martin and Potts, 2004; Mechelmans et al., 2017) levels, is reward hypersensitivity. In our study, we hypothesized that neural indices associated with processing of feedback and rewards, namely, feedback-related delta and theta power, would be positively associated with impulsivity. In contrast to our hypotheses, we failed to find an effect of impulsivity (DF) on these measures. To the best of our knowledge, only one study has investigated feedback-locked delta power in relation to impulsive behavior, and it also did not find this association (Pornpattananangkul and Nusslock, 2016). In contrast, there is multiple evidence showing that feedback-locked theta activity correlates with self-reported impulsivity scores (Kamarajan et al., 2008; Leicht et al., 2013; Andreou et al., 2017) as well as behavioral performance during the DD task specifically (Pornpattananangkul and Nusslock, 2016). The absence of association between impulsivity and reward-related time-frequency measures in our data is likely attributable to the deterministic nature of our task design. In our paradigm, the outcome was known and certain upon choice, thus minimizing or eliminating any reward prediction error. The feedback, therefore, served as a confirmation of an expected event rather than a signal of unexpected gain. As already mentioned, our paradigm, in contrast, did not include loss or probabilistic conditions, as we were focused solely on the processing of rewards (gains) when they were known and expected. Thus, within the limited context of our design, we can only conclude that when reward prediction error is absent, impulsive individuals do not process the mere receipt of the rewards differently from self-controlled ones.

Finally, another critical process that contributes to impulsivity and decision-making, which our study investigated, is how well an individual can tolerate a delay preceding a reward. Accordingly, we assessed neural anticipatory mechanisms by measuring power spectral density (PSD) in the alpha band during anticipation of large rewards and observed increased parieto-occipital alpha PSD during reward anticipation in more impulsive individuals, in line with our hypotheses. In the context of our task design, we suggest that increased alpha PSD during the anticipation may capture a specific aspect of impulsive behavior: irrational delay intolerance. As our instructions discouraged participants from speeding through the experiment, participants with lower DFs chose the smaller rewards despite knowing it was sub-optimal and would not save them time. This suggests that for these individuals, the subjective difficulty of the wait or the immediate attraction of the SS rewards outweighed the rational instruction to maximize points. This interpretation is also in line with research that has consistently linked a decrease in alpha band power to enhanced sustained attention (Bacigalupo and Luck, 2022) and inhibitory gating (Jensen and Mazaheri, 2010; Doesburg et al., 2016; Woodman et al., 2022), which suggests less efficient mechanisms of attentional focusing ability to inhibit irrelevant stimuli. Furthermore, self-controlled individuals maintained this neural signature of focused attention (lower alpha PSD) despite the longer overall duration of their experiment, reinforcing the link between delay tolerance and sustained cognitive engagement. Another potential explanation for individual differences in the alpha band during the anticipation may involve differences in the subjective perception of time, an ability that has been linked to impulsivity (Wittmann and Paulus, 2008; Paasche et al., 2019) and is mediated by alpha oscillations (Rogachev and Sysoeva, 2023). The novelty of our findings relates to the use of experiential DD, as we are the first to show, using electrophysiology, that self-controlled individuals better tolerate delays due to being more alert during waiting, thus linking our findings with existing fMRI studies that used an experiential design (Tanaka et al., 2020) and providing a potential for translational research where hypothetical delays cannot be used (Lukinova et al., 2019).

Taken together, our results provide insights into the electrophysiological correlates of impulsive decision-making using an experiential DD task design and further elaborate on the evidence of less efficient mechanisms of reward anticipation in impulsivity as measured by DD.

## 5 Limitations and future directions

In our data, we were not able to find any electrophysiological measures of reward sensitivity that correlated with impulsivity as measured by DD, which may be partially attributable to the specific design of our experiential task. While ecologically valid for studying waiting, it was not optimized to elicit reward processing driven by reward prediction error, as the feedback-related responses indicated a mere fact of receiving the reward. Additionally, the design and instructions of our task (that the trial count and total duration were fixed), which encouraged participants to collect as many points as possible and intended to prevent too many participants from consistently choosing the SS option to rapidly complete the experiment, led to a substantial number of our participants reaching a ceiling effect and having close to maximum values of DF. This resulted in a low trial count for SS choices and consequently restricted our ability to include reward magnitude in the analysis of reward sensitivity measures and investigate its interaction with DF.

Future research utilizing experiential DD paradigms utilizing EEG should specifically address the design limitations outlined above, potentially by refining task instructions or mechanics to better disentangle reward sensitivity from session-duration strategies and mitigate low trial counts. Additionally, given the prevalence of digital technology among young adults, future work might benefit from incorporating other measures of individual differences, such as self-reported impulsivity scores and digital behavior patterns, which have been linked to delay discounting (van Endert and Mohr, 2020; Cheng et al., 2021; Weinsztok et al., 2021; Pancani et al., 2023).

## 6 Conclusion

In our study, we focused on two processes that contribute to impulsive decision-making in the experiential DD task: rewards processing and reward anticipation. We suggest that impulsive individuals exhibit less efficient mechanisms of rewards anticipation, as indexed by higher alpha band power in the parieto-occipital regions, which may explain their lower tolerance to delays. Additionally, we found no evidence for enhanced rewards processing in more impulsive individuals. Taken together, our results expand the electrophysiological evidence for altered mechanisms of reward anticipation, using the example of an experiential DD task.

## Data Availability

The datasets presented in this study can be found in online repositories. The names of the repository/repositories and accession number(s) can be found in https://github.com/fzakirov/dd_eeg/.
