# Supplementary material for: Atypical reward anticipation in impulsive individuals: evidence from EEG and experiential delay discounting
Source: Front Psychol. 2026 Jan 12;16:1746734. doi: 10.3389/fpsyg.2025.1746734 (PMC12833305; doi:10.3389/fpsyg.2025.1746734)
Supplement: Supplementary file 1 [file Table_1.docx]

***Supplementary Material***


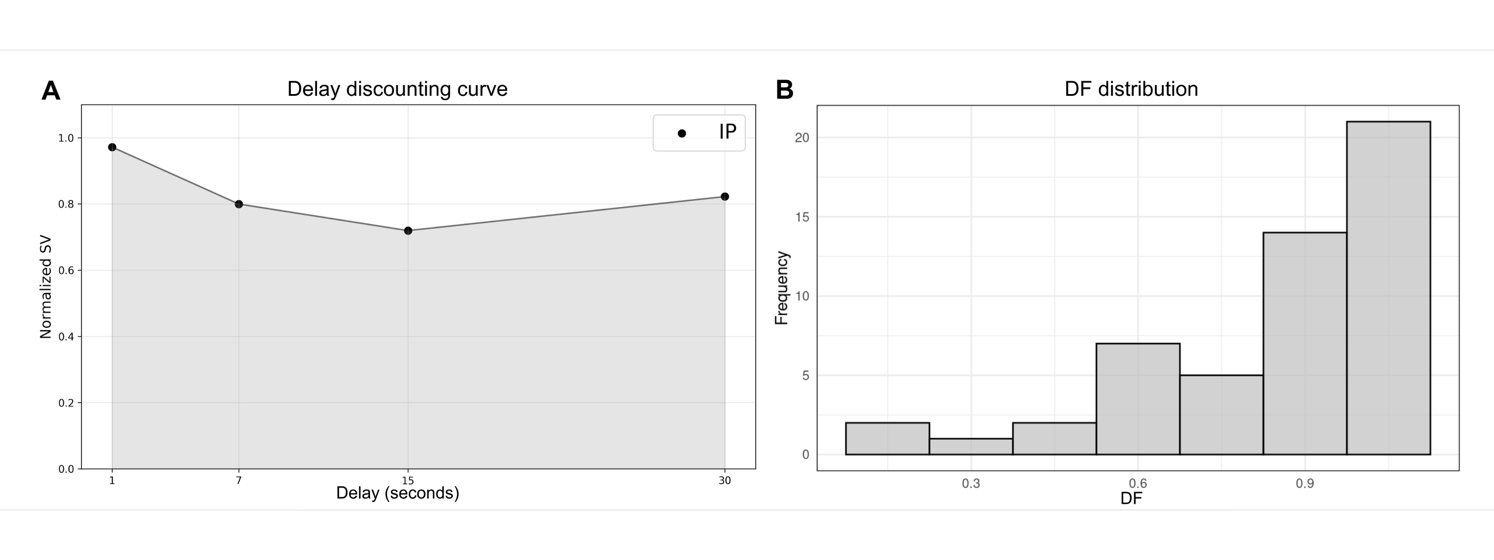


**Supplementary Figure 1.** (A) Delay discounting curve averaged across participants (n = 54). (B) DF distribution (n=54). SV: subjective value, IP: indifference point, DF: discounting factor (see the main text)


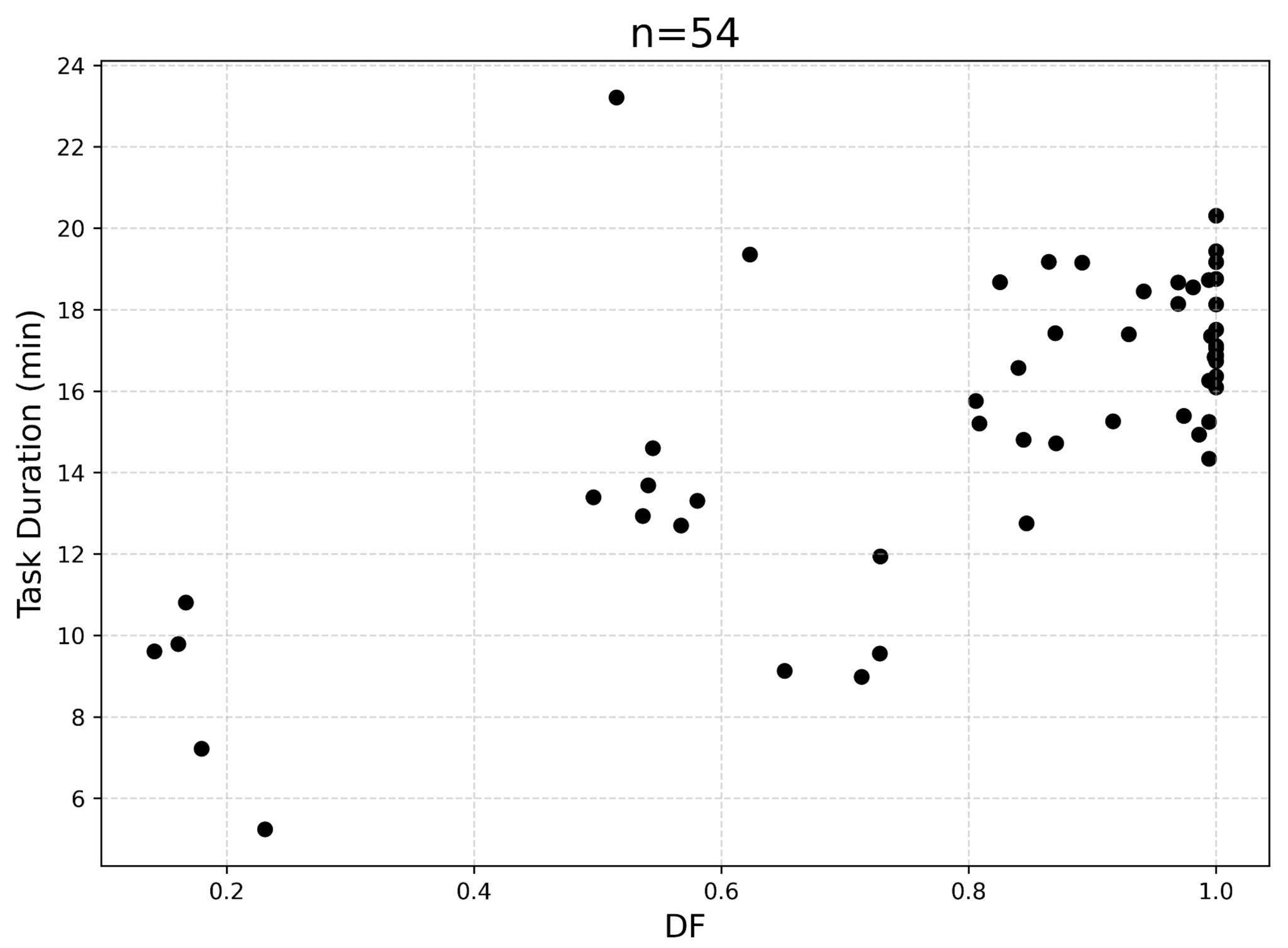


**Supplementary Figure 2.** Relationship between task duration (in minutes) and DF (n = 54). Participants with higher DF spent more time in the experiment (p < .001).


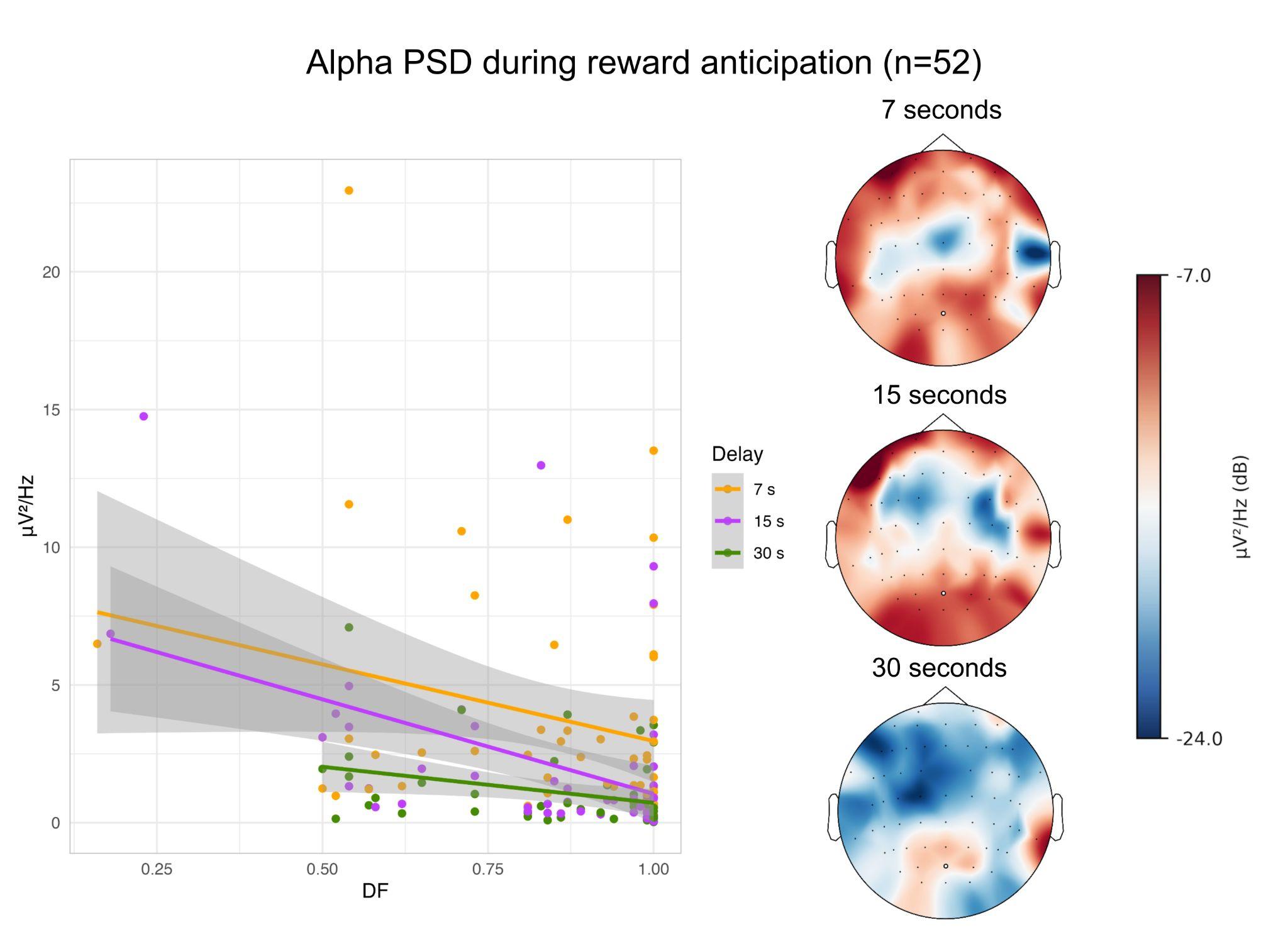


**Supplementary Figure 3.** Parieto-occipital alpha band PSD (n = 52) during the LL anticipation separately for three delay levels (7s, 15s, 30s). Individuals with higher DF (more self-controlled) exhibited reduced alpha PSD during reward anticipation. The regression lines depict the linear model fits, and the shaded region indicates the 95% confidence interval. The PSD values are normalized (dB) to enable the usage of the same scale across three topographies.
